# Intestinal Type of Lung Adenocarcinoma in Younger Adults

**DOI:** 10.1155/2014/282196

**Published:** 2014-03-23

**Authors:** Jelena Stojsic, Milica Kontic, Dragan Subotic, Marko Popovic, Dragana Tomasevic, Jelena Lukic

**Affiliations:** ^1^Service of Histopathology, Clinical Centre of Serbia, Koste Todorovića, 26, 11000 Belgrade, Serbia; ^2^Clinic of Pulmonology, Clinical Centre of Serbia, Koste Todorovića, 26, 11000 Belgrade, Serbia; ^3^Clinic of Thoracic Surgery, Clinical Centre of Serbia, Koste Todorovića, 26, 11000 Belgrade, Serbia; ^4^Medical Faculty, University of Belgrade, Dr. Subotića 8, 11000 Belgrade, Serbia; ^5^Laboratory for Biochemistry and Molecular Diagnostics, “Konzilijum,” Višegradska, 25, 11000 Belgrade, Serbia

## Abstract

Intestinal type of lung adenocarcinoma (ILADC) was initially described by Tsao and Fraser in 1991. Morphology and immunophenotype of ILADC are the same as in colorectal adenocarcinoma. Rectocolonoscopy must be performed to exclude colorectal origin of adenocarcinoma. Colorectal adenocarcinoma claimed to be genetically similar to an ILADC. *Patients*. We describe 24- and 26-year-old patients of both genders who went under surgery because of a lung tumor mass detected on CT scan. ILADC was diagnosed on resected lung specimens. According to positivity of Cytokeratin20, CDX-2, and Villin, respectively, and negativity of Cytokeratin7, TTF-1, Napsin-A, SurfactantB, MUC-1, and MUC-2, respectively, ILADC was diagnosed. KRAS mutation was detected in tumor tissue of the male patient. *Conclusion*. Rectocolonoscopy is the only relevant method for distinguishing the intestinal type of lung adenocarcinoma from metastatic colorectal carcinoma because immunohistochemistry and detection of mutation status are frequently the same in both types of adenocarcinoma. More investigations are needed for further understanding of ILADC in purpose of personalized lung carcinoma therapy particularly introducing detection of mutation status, especially in younger patients.

## 1. Introduction

In the last few decades, adenocarcinoma is the worldwide most common histologic subtype of lung carcinoma. It develops more frequently than any other histologic types of lung carcinoma in no smokers, particular in women. Intestinal type of lung adenocarcinoma ILADC [1, 2] was not mentioned in WHO lung carcinoma classification from 1999 to 2004. In recent years, International Study Group of Lung Carcinoma introduced ILADC in adenocarcinoma classification [3–5], but this variant of lung adenocarcinoma was initially described by Tsao and Fraser in 1991, and it was characterized by a predominant component of malignant tall, stratified columnar, and goblet cells [6].

Immunophenotype of ILADC is the same as in colorectal adenocarcinoma. Malignant cells are positive for Cytokeratin20 and CDX-2 and negative for Cytokeratin7 and TTF-1 [7–9]. Rectocolonoscopy must be performed in order to exclude colorectal origin of adenocarcinoma. Patients reported in the respected literature were older than patients in our study [9].

It is well known that EGFR and KRAS gene mutations act as positive and negative predictors, respectively, of therapeutic response to EGFR targeted therapies in colorectal adenocarcinoma. This tumor claimed to be genetically similar to ILADC [10].

Here we describe 24-year-old and 26-year-old patients, male and female, with ILADC diagnosed on resected lung specimens. Genetic testing was also performed in order to evaluate the occurrence and consequence of EGFR alterations and KRAS mutations in these two patients. Both patients gave individual consents for publishing this report.

## 2. Patients

### 2.1. Surgery

**Figure 1:**
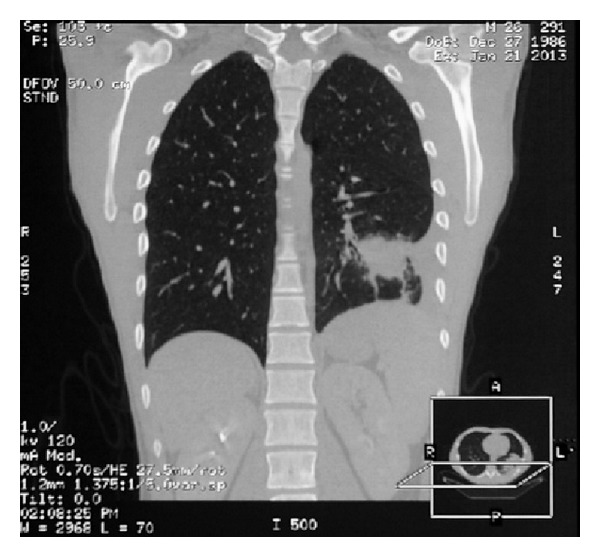
Tumor shadow was revealed in left lower lobe of the lung on chest CT scan.

Both patients went under surgery because of tumor mass detected on CT scan (Figure 1). Resection of parietal pleura and partial resection of the callous 8th rib was performed in the female patient. In male patient, left lower lobectomy with mediastinal lymphadenectomy was performed.

### 2.2. Pathology Findings

After the surgery, tissue samples were fixed in 10% neutral-buffered formalin, paraffin embedded, and hematoxylin-eosin stained.

Massive involving of ILADC of parietal pleura was diagnosed in the female patient. Deposits of adenocarcinoma were found in the thickening part of the 8th rib, measured up to 80 mm. Tumor stage disease was established as T_3_N_0_.

In male patient, ILADC was limited only in lung tissue, measured up to 60 mm, spreading only to intrapulmonary lymph node and without involvement of visceral pleura. Tumor stage disease was established as T_2b_N_1_.

Morphological pattern of malignant tall, stratified columnar, and goblet cells, degree of necrosis, and desmoplastic reaction were similar to any metastatic intestinal adenocarcinoma of colorectal origin.

### 2.3. Immunohistochemistry

Immunohistochemical staining using the avidin-biotin complex and peroxidase methods was performed on 4 mm sections. Monoclonal antibodies were applied according to the manufacturer prescription and they are listed in Table 1. Appropriate negative and positive controls were included.

Immunohistochemical findings are given in Table 2.

**Figure 2:**
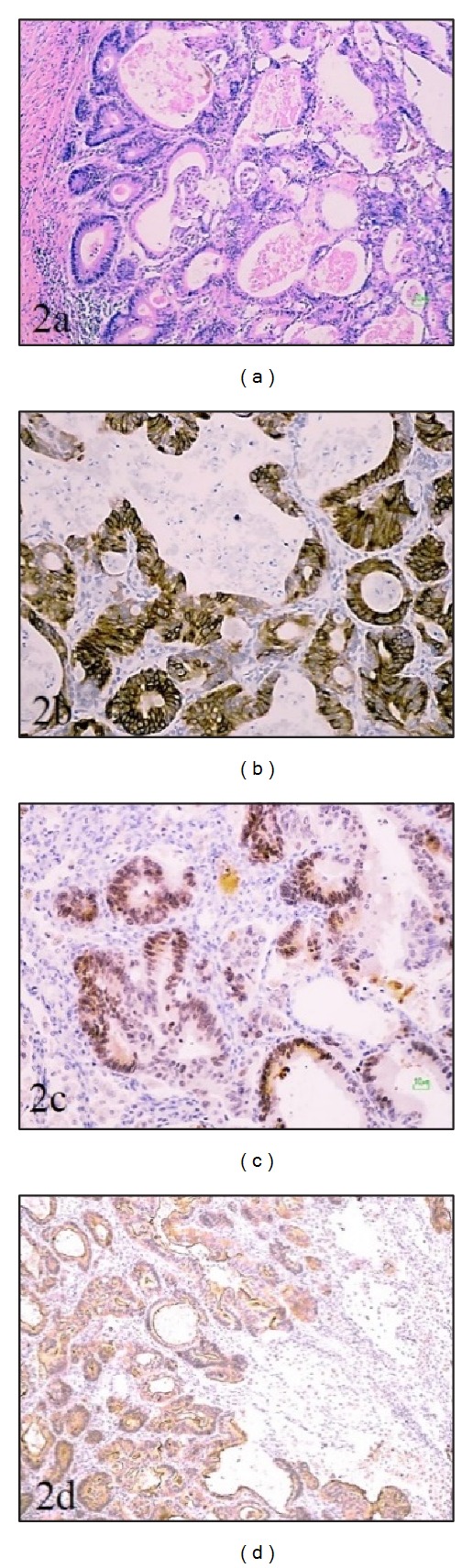
Pathological finding of ILADC in female patient: (2a) intestinal type of lung adenocarcinoma, H&Ex10, and its immunohistochemical profile: positivity of (2b) Cytokeratin 20×20; (2c) CDX-2×20; (2d) Villin ×10.

In both patients, tumor cells expressed the panel of monoclonal antibodies characteristic for ILADC, but not for usual type of lung adenocarcinoma. After immunohistochemistry diagnosis, ILADC was confirmed. Immunohistochemical findings of the female patient are given in Figure 2.

### 2.4. Genetic Findings

DNA from paraffin-embedded lung tumor specimens was prepared from 10 × 30 *μ*m sections after macrodissection. Genomic DNA was isolated after xylene extraction and proteinase K digestion and purified with QiaAmp Minelute Columns. PCR primers were designed to amplify 2. exon of KRAS gene and 18., 19., 20., and 21. exons of EGFR gene. The resulting PCR products were sequenced by using bidirectional dye-terminator fluorescent sequencing. Sequencing fragments were detected via capillary electrophoresis with ABI Prism 3130 DNA Analyzer (Applied Biosystems). The KRAS and EGFR mutation screening was done according to the methods of analysis in the different collaborative centers.

**Figure 3:**
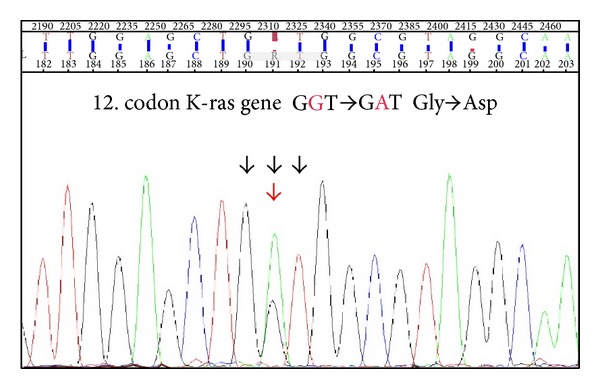
KRAS mutation was detected in male patient.

In male patient, we detected mutation in 2. exon 12. codon of KRAS GGT->GAT, Gly->Asp (Figure 3). Mutations in EGFR gene were not detected in both patients.

### 2.5. Outcome

Colorectal endoscopy was performed in both patients to exclude lung metastasis of colorectal adenocarcinoma.

Both patients are still under frequent controls or chemotherapy, without evidence of recurrence or metastasis.

## 3. Discussion

There are not many reports on ILADC because it is extremely rare variant of lung adenocarcinoma. Yousem reported only 6 intestinal types of lung adenocarcinoma in a review of 430 lung adenocarcinomas diagnosed from 1996 to 2004. In this study, patients ranged from 57 to 82 years old [7]. Our patients were 24- and 26-year-old.

It is difficult to distinguish ILADC from metastatic colorectal adenocarcinoma, according to morphological pattern and immunophenotype. Some reports [7–9, 11] presented coexpression of Cytokeratin7, Cytokeratin20, TTF-1, and MUC-2, without expression of CDX-2. Malignant cells of ILADCs in our report expressed Cytokeratin20, CDX-2, and Villin without expression of MUC-2.

Usual type tumor cells of lung adenocarcinoma frequently express TTF-1, Cytokeratin7, Napsin-A, and any of Surfactants. It is enough for 2 to 3 of these monoclonal antibodies to be expressed for diagnosis of usual lung adenocarcinoma [12, 13].

Activating KRAS mutations represents the most common abnormality of a dominant oncogene in human carcinoma, with specificity and type of mutation varying in relation to tumor type. The rat sarcoma (RAS) genes, including HRAS, KRAS, and NRAS, encode a family of proteins regulating cell growth, differentiation, and apoptosis [14].

KRAS mutations are present in 20–30% of non-small cell lung carcinoma (NSCLC) and occur most commonly in adenocarcinoma histology and life-long smokers. On the other hand, mutations in KRAS are among the critical transforming alterations occurring during colorectal tumorigenesis. 30–40% patients with colorectal carcinomas have these mutations, which occur early in the progression from adenoma to carcinoma. Although in colorectal adenocarcinoma the KRAS mutations are the most useful biomarker for selecting patients who are candidates for treatment with anti-EGFR monoclonal antibodies, its role in NSCLC as prognostic or predictive marker is less defined [15].

The frequency of KRAS mutations in codon 12 or 13 significantly differs between sporadic and hereditary settings: KRAS mutations in codon 12 are more common in sporadic carcinomas, whereas mutations in codon 13 are predominant in hereditary cases, like hereditary nonpolyposis colorectal adenocarcinoma. A good example of the presence of codon 12 mutations in sporadic carcinomas and its association with environmental factors is the predominance of codon 12 KRAS mutations in environmental associated adenocarcinomas, such as lung and colon carcinoma associated with tobacco smoking and bladder carcinoma. The explanation of the differences found between the location of the mutation in sporadic and hereditary tumors could be the fact that sporadic carcinomas may be associated with an increased susceptibility to damage in specific DNA sequences by environmental factors, whereas hereditary tumors occur due to inherited predisposition. KRAS mutations are one of the most commonly occurring oncogene aberrations in human carcinoma, but no specific treatment is currently available [16].

In this report, KRAS mutation was identified in patient with primary pulmonary adenocarcinoma with intestinal differentiation. We suggest the introduction of EGFR and KRAS mutation status analysis in the purpose of personalized lung carcinoma therapy of ILADC.

## 4. Conclusion

Only relevant method to distinguish intestinal type of lung adenocarcinoma from metastatic colorectal carcinoma is rectocolonoscopy, because additional methods such as immunohistochemistry and detection of mutation status are frequently the same in both types of adenocarcinoma. More investigations are needed for further understanding of ILADC. With purpose of personalized lung carcinoma therapy intestinal type of lung adenocarcinoma introducing detection of mutation status is also recommended, especially in younger patients.

## Figures and Tables

**Table 1:** Panel of applied antibodies, their manufacturer, dilution, and reaction.

Number	Antibody and clone	Manufacturer	Dilution	Reaction
1	Cytokeratin7; Clone OV-TL 12/30	Dako	1 : 50	Cytoplasmatic
2	Cytokeratin20; Clone K_s_20.8	Dako	1 : 25	Cytoplasmatic
3	TTF-1; Clone 8G7G3/1	Dako	1 : 50	Nuclear
4	CDX-2; Clone: DAK-CDX2	Dako	1 : 50	Nuclear
5	Napsin-A; Clone: IP64	Novocastra	1 : 400	Punctate cytoplasmatic
6	Villin; Clone 1D2 C3	Dako	1 : 50	Membranous and cytoplasmatic
7	SurfactantB; Clone: 19H7	Novocastra	1 : 25	Cytoplasmatic
8	MUC-1; Clone: Ma695	Novocastra	1 : 100	Cytoplasmatic
9	MUC-2; Clone: Ccp58	Novocastra	1 : 100	Cytoplasmatic

**Table 2:** Immunoreactivity of intestinal lung adenocarcinoma in both patients.

Monoclonal antibody	CK7	CK20	TTF-1	CDX-2	Villin	Napsin-A	MUC-1	MUC-2	SurfactantB
Female	−	+	−	+	+	−	−	−	−
Male	−	+	−	+	+	−	−	−	−

Abbreviations: CK7: Cytokeratin7; CK20: Cytokeratin20; TTF-1: Thyreoid Transcriptive Factor-1.
